# Role of nutritional status and physical activity in the relationship between sleep quality and cardiometabolic proﬁle of children

**DOI:** 10.5935/1984-0063.20200067

**Published:** 2021

**Authors:** Camila Felin Fochesatto, Caroline Brand, Arieli Fernandes Dias, Luiza Naujorks Reis, Jorge Mota, Adroaldo Cezar Araujo Gaya, Anelise Reis Gaya

**Affiliations:** 1 Universidade Federal do Rio Grande do Sul, Physical Education, Physical Therapy and Dance School, Porto Alegre, Rio Grande do Sul, Brazil.; 2 Graduate Program in Health Promotion, Universidade de Santa Cruz do Sul, Santa Cruz do Sul, Rio Grande do Sul, Brazil.; 3 Universidade do Porto, Faculty of Sport, Porto, Portugal.

**Keywords:** Sleep, Obesity, Health Promotion

## Abstract

**Objective:**

To verify if there is an association between sleep quality and cardiometabolic risk factors (CMRF) score of children and the role of nutritional status and physical activity (PA) levels in this relationship.

**Material and Methods:**

Cross- sectional study, with 83 schoolchildren, aged 6 and 11 years, from Porto Alegre, Brazil. PA was assessed with accelerometer and nutritional status through body mass index. Total cholesterol, triglycerides, glucose, and insulin were collected to calculate CMRF score. Parents’ perception of their children’s sleep was evaluated through a question. Generalized linear models were used.

**Results:**

Poor sleep quality was positively associated with CMRF (ß=0.48; CI 95%=0.23;0.73) in relation to those with good sleep quality. A positive association was found in overweight children with poor sleep quality (ß=0.52; CI 95%=0.17;0.86), as well as in inactive children with poor sleep quality (ß=0.58; CI 95%=0.14;1.02) with CMRF.

**Conclusion:**

Poor sleep quality integrated with overweight and physical inactivity influences on CMRF of children.

## INTRODUCTION

Occurrence of cardiometabolic risk factors (CMRF) in childhood and adolescence have been increasing in the last years^1^. This is a worrying reality, since the early development of these factors is related to the appearance of chronic diseases in adult life, such as diabetes mellitus and hypertension^2^. Besides the genetic component, changes that have been occurring in the lifestyle of children and young people have been evidenced as the main modifiable factors associated to CMRF in the childhood^3^. Behaviors such as insufficient sleep and poor sleep quality^4^, physical inactivity and sedentary behavior^5^ are increasingly recurrent in the routine of this population.

Among these behaviors, the role of sleep, including its quantity and quality, seems to be relevant in different perspectives of schoolchildren’s health^6^. Data regarding the amount of sleep are already well established and indicate that not following the recommendations for sleep time (9 to 11 hours) is associated to a reduction of high-density lipoprotein cholesterol (HDL-C), greater adiposity, glucose, and insulin resistance in children and adolescents^6^. On the other hand, evidences concerning sleep quality are limited and conflicting. Whereas Pulido-Arjona et al. (2018)^7^ found an association between sleep quality and lipid profile, Lo et al. (2019)^8^ demonstrated that this relationship was not significant. Furthermore, physical activity (PA) has been indicated as a strategy for changing sleep patterns. In this regard, an intervention program with physical exercise practice during 12 weeks promoted improvement in sleep quality of adolescents^9^. Therefore, there is a complex relationship between different behaviors related to the lifestyle of children.

Based on the assumption that there would be an association between sleep quality and CMRF, we hypothesized that nutritional status and PA level could influence on this relationship. We believe that a combination of unhealthy behaviors may already be related to an increased risk of developing early diseases. What guides this presumption is the fact that the literature demonstrates an independent association between these aspects^1,10^, however, without establishing the influence on the relationship between sleep quality and CMRF.

From that, the relevance of the present study is justified in the perspective of filling a gap in the literature regarding the relationship between sleep quality and CMRF in children, since most studies refer to sleep time^6^. In addition, understanding the role of nutritional status and PA level is important, since metabolic health is influenced by multiple aspects and a wider understanding of how these relationships occur is necessary. Therefore, the aim of the present study was to verify if there is an association between sleep quality and CMRF score in children and the role of nutritional status and PA level in this relationship.

## MATERIAL AND METHODS

This is a cross-sectional study, with a quantitative approach. The sample consisted of 83 schoolchildren (40 boys) aged between 6 and 11 years (mean 8.72±1.43), from a public elementary school in Porto Alegre, Brazil, selected by convenience criteria. The definition of this school is justified by presenting an agreement with the research institution. All children from the first to the fifth grade were invited to participate in the study. The parents/guardians signed the free and informed consent form and the children signed the assent form. The study included children who agreed to participate, presented parental authorization and performed all assessments. In addition, the present study was approved by the Human Research Ethics Committee of the Universidade Federal do Rio Grande do Sul under N° 2.611.180.

The minimum number of subjects in the sample was calculated posteriori using the G*Power software version 3.1. A weak effect size (f2=0.13) and an alfa of 0.05 were used. Thus, a statistical power of 0.77 was reached. Linear regression models were tested with four predictors in each model.

To evaluate the parents’ perception of their children’s sleep quality, the parents attended a meeting, and for those who were unable to attend, an individual meeting was scheduled at the school. Sleep quality was verified through a question addressed to parents: “Do you consider your child’s sleep quality to be”. The response options were: “very good”, “good”, “bad” or “very bad”. For analysis purposes, responses were categorized as “good” (very good + good) and “bad” (bad + very bad).

Anthropometric assessments and procedures for placing and removing the accelerometers were held at the school by a team of trained researchers. Height and body mass were assessed following the PROESP-Br procedures^11^. Height was verified using a tape measure fixed to the wall and extended from the bottom up, with the children kept in an upright position, with their feet and trunk leaning against the wall. This measurement was recorded in centimeters to one decimal place. Body mass was measured using a digital scale, accurate to 500 grams and recorded in kilograms to one decimal place. Children should be barefoot, wearing light clothing, standing with their arms close to their body. From that, body mass index (BMI) was calculated by dividing body mass (kg) by height (m) squared. Values of nutritional status were categorized as underweight, normoponderal, overweight and obese according to Conde and Monteiro (2006)^12^ and for analysis purposes, they were classified as “normoponderal” (underweight + normoponderal) and “overweight” (overweight + obese).

For the calculation of somatic maturity, sitting height (SH) and leg length (LL) were evaluated. In the first, a backless bench was positioned on a wall and a measuring tape was fixed at the height of its seat, from the bottom up. Then, the child was asked to sit with his/her whole back against the wall and with the help of a square positioned transversely to the head of the student, the SH was verified, in centimeters. Moreover, LL was determined by subtracting SH from the total height of the child (both in centimeters). After that, the procedures described by Mirwald et al. (2002)^13^ were applied, which consist in determining the status of somatic maturity through the identification of the distance, in years, that the individual is in relation to the peak height velocity, using the interaction between age and anthropometric variables of height, weight, SH and LL based on the following equations: for boys, somatic maturity = $-29.769+0.0003007\;\left(\mathrm{LL}*\mathrm{SH}\right)-0.1177\;\left(\mathrm{age}*\mathrm{LL}\right)+0.01639\;\left(\mathrm{age}*\mathrm{SH}\right)+0.445\;\left(\mathrm{weight}/\mathrm{height}\right);$ ; for girls, somatic maturity = $-16.364+0.0002309\;\left(\mathrm{LL}*\mathrm{SH}\right)+0.006277\;\left(\mathrm{age}*\mathrm{LL}\right)+0.179\;\left(\mathrm{age}*\mathrm{SH}\right)+0.0009428\;\left(\mathrm{weight}/\mathrm{height}\right).$

PA levels was measured with an ActiGraph accelerometer (wActiSleep-BT Monitor), which was placed on the child’s waist on an elastic belt, on the right side of the mid-axillary line. Participants were instructed to use the device for seven consecutive days. The equipment should be maintained throughout the day and should only be removed when performing any aquatic activities. Children were advised concerning the proper use of the device and parents received information through messaging applicative. The minimum amount of accelerometer data considered acceptable for analysis purposes was four days (including at least one weekend day), with at least 10 hours/day of usage time. After the last day of accelerometer usage, the research team went to the school to remove the accelerometers and to verify that the data was complete, using the Actilife software (ActiGraph®, version 5.6, USA). Data were collected at 80Hz sampling rate, downloaded in periods of one second and aggregated for periods of 15 seconds. To estimate the minutes in moderate to vigorous physical activity (MVPA), the cut-off point proposed by Evenson et al. (2008)^14^ in 15-second periods was used. Then, mean daily MVPA for each child was calculated and categorized into active (≥60min/day) and inactive (<60min/day), according to the World Health Organization^15^.

Blood samples were also carried out at school, by nurses with experience in children, after 12 hours of fasting, with disposable materials. For transportation to the laboratory, the samples were stored in a thermal box, which allowed the maintenance of the proper temperature. Blood samples were centrifuged, and then plasma and serum were aliquoted and frozen at -80 °C until analysis. Total cholesterol, high-density lipoprotein (HDL-C), triglycerides and glucose concentrations were analyzed by the automated colorimetric method (Cobas C111, Roche, Basel, Switzerland). Glucose was determined using an automated analyzer (Cobas C111, Roche Diagnostics, Basel, Switzerland). Insulin concentrations were determined by ELISA using commercial kits (DRG International, Springfield, USA).

CMRF score was calculated for each participant, using the following formula:

Cardiometabolic risk factors = z-cholesterol total + z-HDL-C + z-triglycerides + z-glucose + z-insulin. HDL-C value was multiplied by -1, as it is inversely associated to cardiometabolic risk.

Data were analyzed through descriptive statistics, presenting mean and standard deviation of all stuwdy variables for the total sample and stratified by sex. Data normality was tested through Shapiro-Wilk test. To verify the difference between boys and girls, independent t-test analysis was used. Thereafter, linear regression models were used for the following analyses: 1) association of sleep quality with z-score of cardiometabolic risk factors (crude model); 2) same association as model 1, adjuster for somatic maturity and gender. To determine the role of nutritional status and PA level, the analyses were stratified into normoponderal and overweight, active and inactive. Then, the interactions of sleep quality X normoponderal and overweight with CMRF score were tested, as well as sleep quality X active and inactive with CMRF score. All analyses were performed using SPSS program version 20.0, *p*<0.05 was considered significant.

## RESULTS

Table 1 presents the sample characteristics stratified by sex. There was a difference between boys and girls for MVPA (t=2.24; *p*=0.02), somatic maturity (t=-7.44; *p*<0.001) and HDL-C (t=2.92; *p*<0,001). In addition, the prevalence of children with poor sleep quality was 25% for boys and 26.5% for girls.

**Table 1.** Sample characteristics stratified by sex.

Variables		Boys		Girls	
	n	Mean (SD)	CI 95%	Mean (SD)	CI 95%
Age (years)	83	8.73 (1.46)	8.27; 9.18	8.72 (1.42)	8.28; 9.14
Body mass (kg)	83	34.89 (9.42)	32.25; 37.84	37.09 (12.21) *	33.52; 41.04
Height (m)	81	1.36 (0.08)	1.34; 1.39	1.36 (0.10)	1.33; 1.39
BMI (Kg/m^2^)	81	18.41 (3.81)	17.29; 19.60	19.52 (4.34)	18.21; 20.80
MVPA (min/day)	57	64.89 (27.00)	55.25; 74.94	52.07 (14.36) *	46.43; 56.98
Total sleep (hours)	65	9.25 (1.19)	8.87; 9.68	9.06 (1.52)	8.56; 9.58
SM (years)	79	-3.70 (0.99)	-4.02; 13.40	-1.40 (1.65) *	-1.92; -0.88
TC (mg/dL)	83	152.70 (25.90)	145.47;161.47	144.17 (27.41)	136.84;153.11
TG (mg/dL)	83	73.11 (32.57)	63.90; 83.64	82.59 (36.44)	72.05; 93.45
HDL-C (mg/dL)	83	47.61 (9.69)	44.65; 50.72	42.09 (7.27) *	39.95; 44.50
Insulin (uU/mL)	83	13.69 (10.56)	10.74; 17.09	13.15 (8.81)	10.73; 15.97
Glucose (mg/dL)	83	92.61 (4.98)	91.02; 94.13	91.80 (7.02)	89.62; 93.83

BMI: Body mass index; MVPA: Moderate to vigorous physical activity; SM: Somatic maturity; TC: Total cholesterol; TG: Triglycerides; HDL-C: High-density lipoprotein cholesterol; SD: Standard deviation;

* Independent t-test for differences between boys and girls (p≤0.05).

Table 2 presents the associations between sleep quality and CMRF score in crude values and adjusted for somatic maturity and sex. A significant association was observed in both models. There was a positive association between poor sleep quality and CMRF score.

**Table 2.** Association between sleep quality and cardiometabolic risk factors score in children.

		Cardiometabolic risk factors score	
	β	CI 95%	p
**Model 1**			
Sleep quality	1		
Poor	0.54	(0.10; 0.99)	0.01
Model 2 *			
Sleep quality			
Good	1		
Poor	0.48	(0.23; 0.73)	<0.001

* Adjusted for somatic maturity and gender.

**Figure 1:**
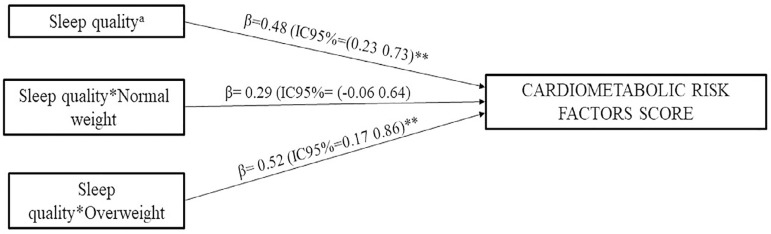
Role of nutritional status in the relationship between sleep quality and cardiometabolic risk factors score. aReference category: Good; *interaction; All analyses were adjusted for somatic maturity and gender; **: p<0.005.

The association between sleep quality and CMRF score in children and the interaction between sleep quality and nutritional status are shown in Figure 1. The results indicated a significant positive association between sleep quality and CMRF score only in overweight children, that is, those children with poor sleep quality and high BMI have a greater CMRF score.

**Figura 2:**
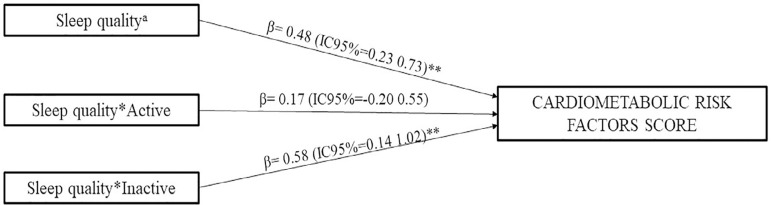
Role of physical activity level in the relationship between sleep quality and cardiometabolic risk factors score. aReference category: good; *interaction; All analyses were adjusted for somatic maturity and gender.

Figure 2 shows the association between sleep quality and CMRF score and the interaction between sleep quality and PA level. A significant positive association was found between sleep quality and CMRF score in inactive children, indicating that poor sleep quality and low MVPA levels are related to a higher CMRF score.

## DISCUSSION

The main results of the present study demonstrated that poor sleep quality was positively associated with CMRF score. In addition, this result was only observed in overweight and inactive children, indicating that those children with poor sleep quality along with high BMI or inactive have a greater CMRF score. Nutritional status and PA level interact with sleep quality exerting negative implications on CMRF score.

Sleep quality influence on CMRF has been recently explored in the literature, especially concerning the pediatric population. Corroborating the results of the present study, Pulido-Arjona et al. (2018)^7^ observed that children who have trouble sleeping, such as difficulty falling asleep, daytime sleepiness and nocturnal awakenings presented lower HDL-C concentrations and greater triglycerides concentrations. Similarly, a cohort study developed with schoolchildren aged 8 to 12 years demonstrated that poor sleep quality was associated with higher triglycerides concentrations^16^.

From these findings, the importance of a good sleep quality for metabolic health benefits is highlighted. However, between 25% and 50% of children present sleep-related problems^4^, which is in accordance with our results. In addition, considering that sleep is a multidimensional construct, besides sleep quality, its quantity must also be taken into account. Regarding sleep duration, evidences are more established and indicate that short and long sleep durations are associated with several risk factors for cardiometabolic diseases^17^.

Another aspect that has been widely investigated is the determining role of obesity in cardiometabolic diseases^1^. From that, we sought to understand its influence on the relationship between sleep quality and CMRF score. Our findings indicated that sleeping poorly and being overweight, when combined increase the cardiometabolic risk. So, the present study brings new information in this respect, since sleep quality and nutritional status have been independently addressed in the literature and in studies with adults^17,18^.

Vicente-Herrero et al. (2017)^18^ found an association between poor subjective sleep quality and increased cardiometabolic risk attributed to obesity in adults, which corroborates our study, evidencing that the concern with such behaviors should start in childhood. Possible mechanisms related to the influence of sleep quality are the increased appetite through loss or reduction of orexigenic activity inhibition in the hypothalamic area and the hypothalamic activity itself (appetite regulation)^19^.On the other hand, regular physical activity is considered a protective factor for cardiometabolic diseases, besides being associated to a better sleep pattern^9^. In this way, our results demonstrated that PA level influences on the relationship between sleep quality and CMRF score. Therefore, being active and sleeping well are fundamental behaviors for good metabolic health, as it is speculated that physical exercise is capable of modulating cytokines, which in turn can influence neural actions, thermoregulation, food intake, sleep, and behavioral patterns^10^.

Considering the abovementioned aspects, we emphasize that the lifestyle habits adopted in childhood are determinant for good health. Children that are more active tend to have better sleep quality^9^, as well as an appropriate body composition^20^. Based on this assumption, it is relevant to address how the relationship between different variables that are interconnected can influence on CMRF.

To our knowledge, this is one of the first studies aimed at investigating the role of nutritional status and PA level in the relationship between sleep quality and a score of CMRF in children. Furthermore, when it comes to sleep patterns, most studies consider sleep time. Therefore, our study brings new evidence that the quality of this behavior, although indirectly assessed, is also crucial for health. On the other hand, some limitations must be mentioned. There are many confounding factors that can influence the relationships addressed, such as eating habits and socioeconomic status. The study design does not allow a cause-effect relationship and the measurement of sleep quality occurred indirectly, which considered the parents’ perception regarding their children’s behavior, leading to underestimate or superestimate sleep quality. Thus, it is suggested that further studies contribute to the perspective of using objective instruments to evaluate sleep quality, in addition to including larger samples.

In conclusion, poor sleep quality of children was positively associated with CMRF score. Moreover, this association only occurred in overweight and inactive children, which suggests a protective factor of physical activity and a negative influence of overweight and obesity. It is important to highlight that the variables considered in the present study are modifiable behaviors. Therefore, interventions aimed at improving body composition, increasing physical activity levels and, consequently, sleep quality should be developed in order to promote health in children.
